# The complete mitochondrial genomes of the Fenton′s wood white, Leptidea morsei, and the lemon emigrant, Catopsilia pomona

**DOI:** 10.1093/jis/14.1.130

**Published:** 2014-10-01

**Authors:** Juan-Juan Hao, Jia-Sheng Hao, Xiao-Yan Sun, Lan-Lan Zhang, Qun Yang

**Affiliations:** 1 College of Life Sciences, Anhui Normal University, 241000 Wuhu, China; 2 State Key Laboratory of Palaeobiology and Stratigraphy, Nanjing Institute of Geology and Palaeontology, Chinese Academy of Sciences, Nanjing 210008, China

**Keywords:** mitochondrial genome, Pieridae, phylogenetic analysis

## Abstract

The complete mitochondrial genomes of *Leptidea morsei* Fenton (Lepidoptera: Pieridae: Dis-morphiinae) and *Catopsilia pomona* (F.) (Lepidoptera: Pieridae: Coliadinae) were determined to be 15,122 and 15,142 bp in length, respectively, with that of *L* . *morsei* being the smallest among all known butterflies. Both mitogenomes contained 37 genes and an A+T-rich region, with the gene order identical to those of other butterflies, except for the presence of a tRNA-like insertion, *tRNA ^Leu^* (UUR), in *C* . *pomona* . The nucleotide compositions of both genomes were higher in A and T (80.2% for *L* . *morsei* and 81.3% for *C* . *pomona* ) than C and G; the A+T bias had a significant effect on the codon usage and the amino acid composition. The protein-coding genes utilized the standard mitochondrial start codon ATN, except the *COI* gene using CGA as the initiation codon, as reported in other butterflies. The intergenic spacer sequence between the *tRNA ^Ser^* (UCN) and *ND1* genes contained the ATACTAA motif. The A+T-rich region harbored a poly-T stretch and a conserved ATAGA motif located at the end of the region. In addition, there was a triplicated 23 bp repeat and a microsatellite-like (TA) _9_ (AT) _3_ element in the A+T-rich region of the *L. morsei* mitogenome *,* while in *C* . *pomona,* there was a duplicated 24 bp repeat element and a microsatellite-like (TA) _9_ element. The phylogenetic trees of the main butterfly lineages (Hesperiidae, Papilionidae, Pieridae, Nymphalidae, Lycaenidae, and Riodinidae) were reconstructed with maximum likelihood and Bayesian inference methods based on the 13 concatenated nucleotide sequences of protein-coding genes, and both trees showed that the Pieridae family is sister to Lycaenidae. Although this result contradicts the traditional morphologically based views, it agrees with other recent studies based on mitochondrial genomic data.

## Introduction

The animal mitochondrial genomes (mitogenomes) are usually circular molecules of 14-19 kb in size, containing 37 genes (including 13 protein-coding genes, two rRNA genes, and 22 tRNA genes) and a non-coding A+T-rich region that regulates the transcription and replication of the mitogenome ( Clayton 1992 ). Due to its simple and compact structure, fast evolutionary rate, and maternal inheritage, it has been used frequently in the studies of population genetics, molecular evolution, phylogenetics, phylogeography, and evolutionary biology ( Simon et al. 1994 ). In recent years, as the DNA sequencing technology has been progressing rapidly, more and more complete animal mitogenome sequences have been determined. To date, more than 240 complete or near-complete mitochondrial DNA sequences have been identified from insects, including 67 from lepidopterans. Of these, the available sequences are mainly from six superfamilies (Bombycoidea, Geometroidea, Papilionoidea, Noctuoidea, Tortricoidea, and Pyraloidea). In total, 33 of these lepidopteran mitogenomes are from butterflies ( Table 1 ).

**Table 1.**
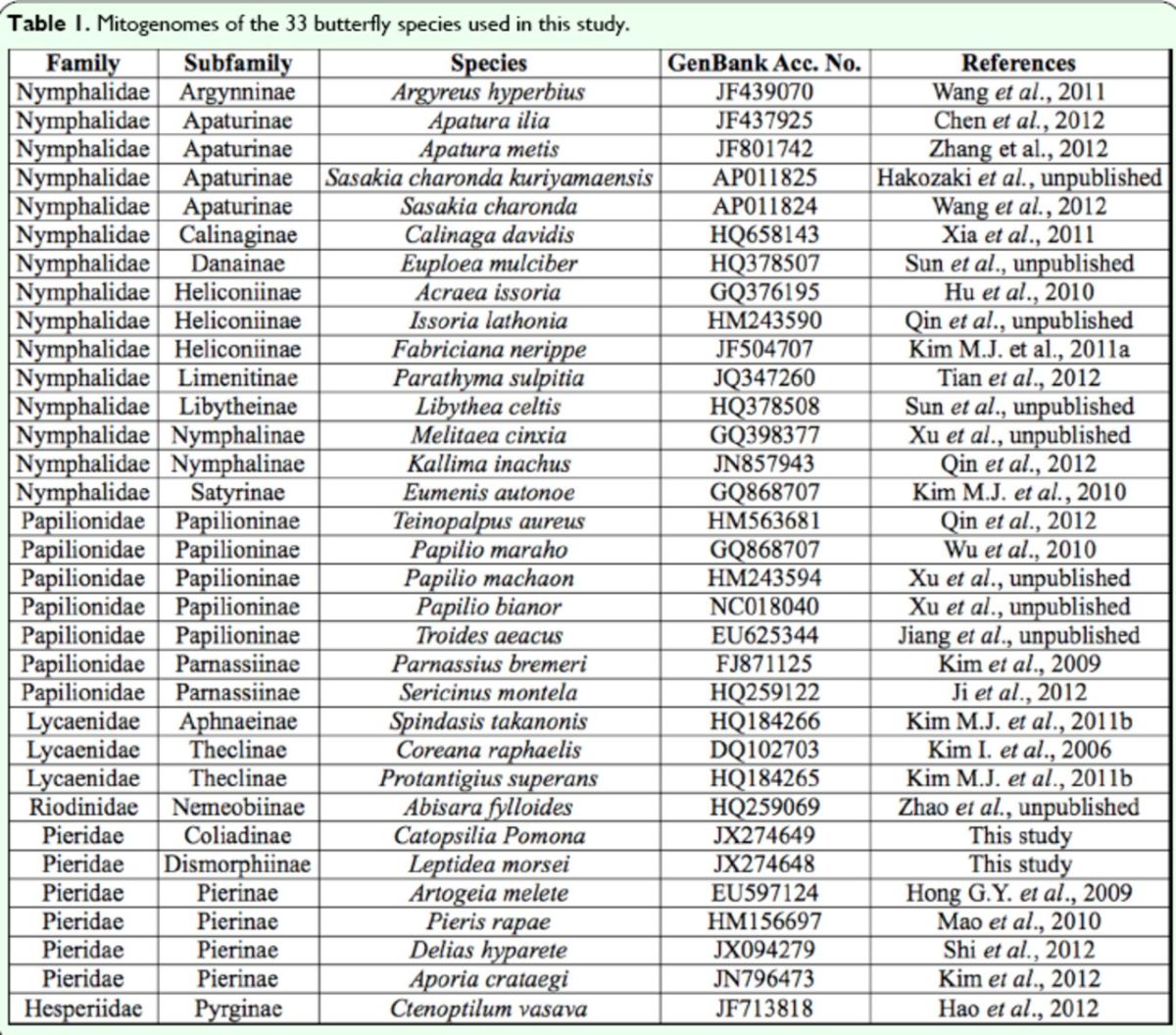
Mitogenomes of the 33 butterfly species used in this study.

Pieridae is one of the largest families of Papilionoidea, containing 76 genera and approximately 1,100 species worldwide, mostly distributed in tropical Africa and Asia ( Courtney 1986 , Watt et al. 1996 , Brunton 1998 , Stavenga et al. 2004 , Kemp et al. 2005 ). Their adults are generally of medium size and typically white, orange, and yellow in color ( Chapman 1895 ). Taxonomically, they are currently divided into four subfamilies (Dis-morphiinae, Pierinae, Coliadinae, and Pseudopontiinae). In addition, phylogenetically, they may stand as a key group to clarify the intra-familiar butterfly relationships. For example, they were traditionally considered to be the sister to the Papilionidae ( Ehrlich 1958 , Scott 1985 ). However, more and more evidence indicated that they were sister to the grouping of (Nymphalidae (Riodinidae, Lycaenidae)) ( Kristensen 1976 , de Jong et al. 1996 , Weller et al. 1996 , Ackery et al. 1999 , Wahlberg et al. 2005 ) or sister to (Riodinidae + Lycaenidae) ( Kim, M. J. et al. 2010 , Chai et al. 2012 ). To our dissatisfaction, up to the present, only four mitogenomes of pierid species *(Artogeia melete* [Menetries], *Pieris rapae* [L.] *, Delias hyparete* [L.] *,* and *Aporia crataegi* [L.]) are available, and thus more pierid mitogenome data are needed to enrich the taxon sampling for use in phylogenetic studies.

The Fenton’s wood white, *Leptidea morsei* Fenton, and the lemon emigrant, *Catopsilia pomona* (F.), are the two representative species of the family Pieridae. *Leptidea morsei* is distributed mainly throughout Europe, Siberia, Ussuri, Korea, northern China, and Japan. It is found occasionally in damp, grassy vegetation at the sunny edges of woods, as well as in grassy woodland. Its larvae feed on peas, and adults are seen twice per year from April to May and June to July. *Catopsilia pomona* is ubiquitously distributed from areas of southeast Asia (Sikkim, Malaysia, Philippines) to Australia. It is found often in secondary forests, along river courses, and even in the hot arid deserts throughout the year. Its colors are usually variable, chiefly lemon-yellow with an apical black margin ( Rienks 1985 ).

In this study, we determined and analyzed the complete mitogenome sequences of these two pierid species and compared these sequences with those of other butterfly species available to clarify the phylogenetic relationships among the main butterfly lineages. The new sequence data will provide valuable information for the studies of lepidopteran comparative genomics, molecular evolution, and other relevant areas.

## Materials and Methods

### Sample collection

Adult individuals of *L* . *morsei* and *C* . *pomona* were collected from Shanxi and Hainan Provinces, China, in August 2008 and July 2009, respectively. After sample collection, the fresh materials were placed into 100% ethanol immediately for DNA fixation and stored at -20°C until used for genomic DNA extraction.

### DNA extraction and amplification by PCR

Total genomic DNA of *L* . *morsei* and *C* . *pomona* was extracted from the thoracic muscle of an adult individual by using the glass bead method after Hao et al. (2005). Insect universal primers were used for the amplification of the *COI* , *CytB* , *16S rRNA* , and *12S rRNA* genes ( Simon et al. 1994 ). Primers for the *ND2* , *ND4* , *COIII* , and *ND5* amplification were designed via the alignment of the respective sequences from all the butterflies available by using Clustal X1.8 and Primer Premier 5.0 softwares ( Thompson et al. 1997 , Singh et al. 1998 ). Seven long fragments ( *COI* – *COIII* , *COIII* – *ND5* , *ND5* – *CytB* , *CytB* – *16S* , *16S* – *12S* , *12S* – *ND2* , *ND2* – *COI* ) were amplified via long PCR using Takara LA Taq™ (Takara Co., www.takara-bio.com ). The long PCR conditions were as follows: an initial denaturation at 95°C for 5 min, followed by 30 cycles of denaturation at 95°C for 50 sec, annealing at 50–55°C (depending on primer pairs) for 50 sec, and elongation at 68°C for 150 sec during the first 15 cycles and then an additional 5 sec per cycle during the last 15 cycles, and a final extension at 68°C for 10 min. All PCR fragments were sequenced directly in both strands after purification with the QIA quick PCR Purification Kit (QIAGEN, www.qiagen.com ), except for the *12S* – *ND2* fragment of *L. morsei* , which was sequenced after cloning. All of the long PCR fragments were sequenced by using the primer walking strategy.

### Sequence analysis

The raw sequences from the overlapping fragments were proofread and assembled in BioEdit version 7.0 ( Hall 1999 ). Protein-coding genes, rRNA genes, and A+T-rich regions were determined via the alignment of the sequences by using Clustal X1.8 software ( Thompson et al. 1997 ). The nucleotide sequences of the protein-coding genes were translated based on the invertebrate mtDNA genetic code. The tRNAs were identified by tRNAscan-SE software version 1.21 ( Lowe and Eddy 1997 ). The putative tRNAs that could not be found by tRNAscan-SE were confirmed by sequence comparisons between the Pieridae and other butterfly tRNAs. Nucleotide composition and codon usage were calculated by using MEGA5.1 software ( Kumar et al. 2004 ), and the tandem repeats in the A+T-rich regions were predicted by the Tandem Repeats Finder available online ( http://tandem.bu.edu/trf/trf.html ) ( Benson et al. 1999 ). Sequence data were deposited in the GenBank database under the accession numbers JX274648 for *L. morsei* and JX274649 for *C. pomona* .

### Phylogenetic analysis

For the phylogenetic analysis, 13 concatenated nucleotide sequences of protein-coding genes of 33 available butterfly mitogenome sequences (two newly sequenced in this study and 31 extracted from GenBank, Table 1 ) were aligned by using Clustal X1.8 ( Thompson 1997 ). The phylogenetic trees were then reconstructed with the maximum likelihood and Bayesian inference methods using the moth species *Adoxophyes honmai* Yasuda (Lepidoptera: Tortricidae) (GenBank accession number DQ073916) as the outgroup.

In the maximum likelihood and Bayesian inference analyses, the third position of all the codons was excluded, and the best fitting substitution model GTR + I + G ( Lanave et al. 1984 ) was selected via a comparison of Akaike Information Criterion scores ( Akaike 1974 ), calculated by using the Modeltest software version 3.7 ( Posada and Crandal 1998 ). The maximum likelihood analyses were conducted in PAUP version 4.0b8 ( Swofford 2002 ) under the following conditions: tree searching by TBR (tree bisection and reconnection) branch swapping (10 random-addition sequences); specifying the number of substitution rate categories as four; and using a BIONJ distance-based tree as the starting tree. The confidence values of each node of the maximum likelihood tree were evaluated via the bootstrapping test with 1,000 iterations. Bayesian analyses were performed by using the program MrBayes 3.1 ( Huelsenbeck and Ronquist 2001 ). Two independent runs of four incrementally heated MCMC chains (one cold chain and three hot chains) were simultaneously run for one million generations in all datasets. Each set was sampled every 100 generations with a burn-in of 25%, and when the average standard deviation of split frequencies was less than 0.01, stationarity was considered to be reached. The confidence values of the Bayesian inference tree were presented as the Bayesian posterior probabilities in percentages.

## Results and Discussion

### General features

**Figure 1.**
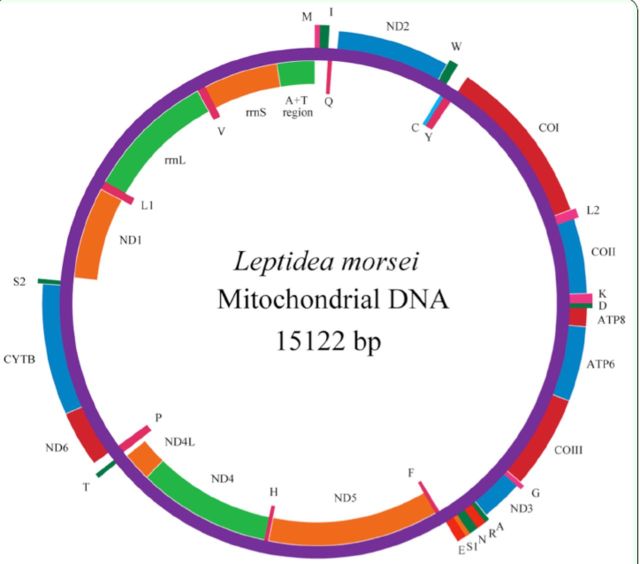
Map of the circular mitochondrial genome of *L morsei.* Genes encoded in the H-strand (clockwise orientation) are colored in red or blue. Genes encoded in the L-strand (anticlockwise orientation) are colored in orange or green. Abbreviations for the genes: *COI-III* for cytochrome oxidase subunits, *CYTB* for cytochrome b, and *ND1-6* for NADH dehydrogenase components. tRNAs are denoted as one-letter symbols according to the IUPAC-IUB single-letter amino acid codes. High quality figures are available online.

The complete mtDNA sequences of *L. morsei* and *C. pomona* were 15,122 and 15,142 bp in length, respectively, with that of *L. morsei* being the shortest among all known sequences of butterfly species ( Table 2 ). Each genome was composed of the typical 13 protein-coding genes, 22 tRNA genes, two rRNAgenes, and one major non-coding A+T-rich region. The gene order was identical to that of other sequences of butterflies but different from that found in the ancestral insects with respect to the location of *tRNA ^et^ .* That is to say, the *tRNA ^et^* was located between the control region and *tRNA* , giving the derived control region (CR)-Met (M)-Ile (I)-Glu (Q) arrangement instead of that of the insect ground plan CR-I-Q-M ( Fig. 1 ). The total sizes of protein-coding genes, rRNAs, and tRNAs were all well within the corresponding ranges of those found in other butterfly species ( Table 2 ). The size proportions of coding genes to the whole genome of these two and four other pierid species were slightly higher than those of butterflies of other families ( Table 3 ). By contrast, their non-coding sequences, including intergenic spacers and the A+T-rich region, were slightly shorter than those of other family taxa. The A+T-rich region of *C. pomona* was the shortest, whereas that of *Papilio maraho* Shiraki & Sonan (Lepidoptera: Papilionidae) was the longest ( Table 4 ). The majority strand coding nine protein-coding genes and 14 tRNAs were 7,804 and 7,836 bp, respectively, whereas the minority strand coding four protein-coding genes, eight tRNAs, and two rRNA genes were 6,901 and 6,934 bp, respectively, for the two pierid species.

**Table 2.**
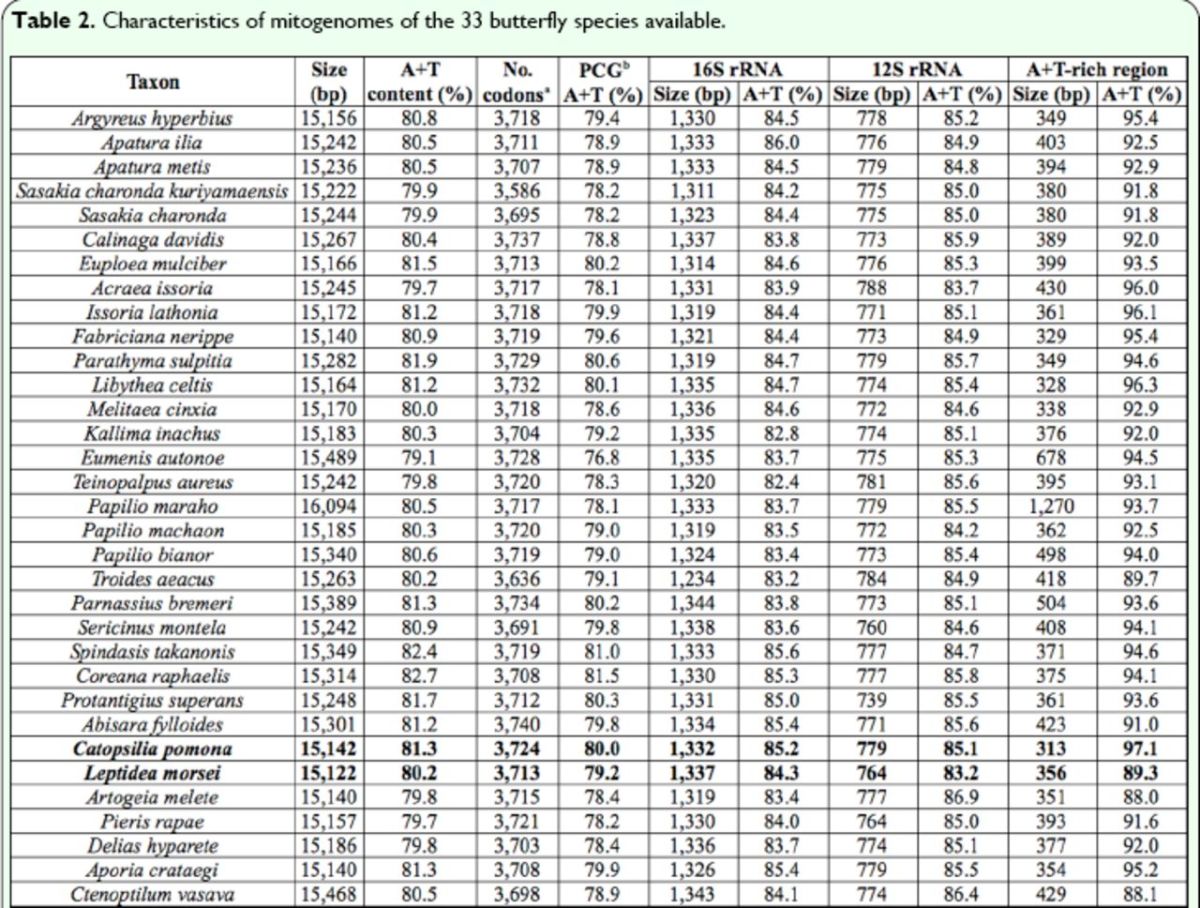
Characteristics of mitogenomes of the 33 butterfly species available.

*a* Termination codons were excluded from total codon count.

*b* Protein coding genes.

**Table 3.**
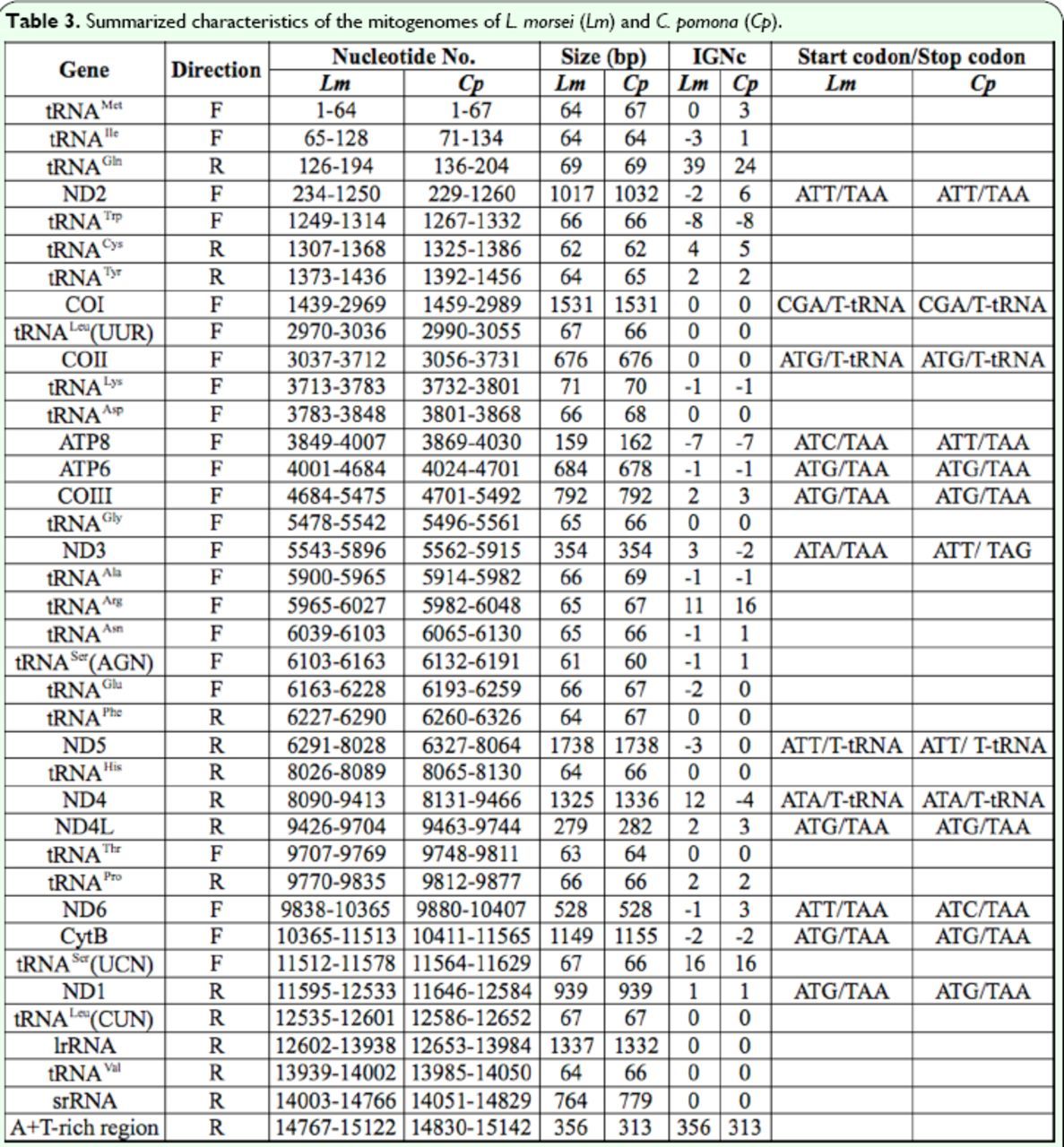
Summarized characteristics of the mitogenomes of *L. morsei* ( *Lm* ) and *C. pomona* ( *Cp* ).

IGNc: intergenic nucleotide length,the positive number indicates interval nucleotides (base pairs) between genes, while the negative number indicates the overlapped nucleotides (base pairs) between genes.

**Table 4.**
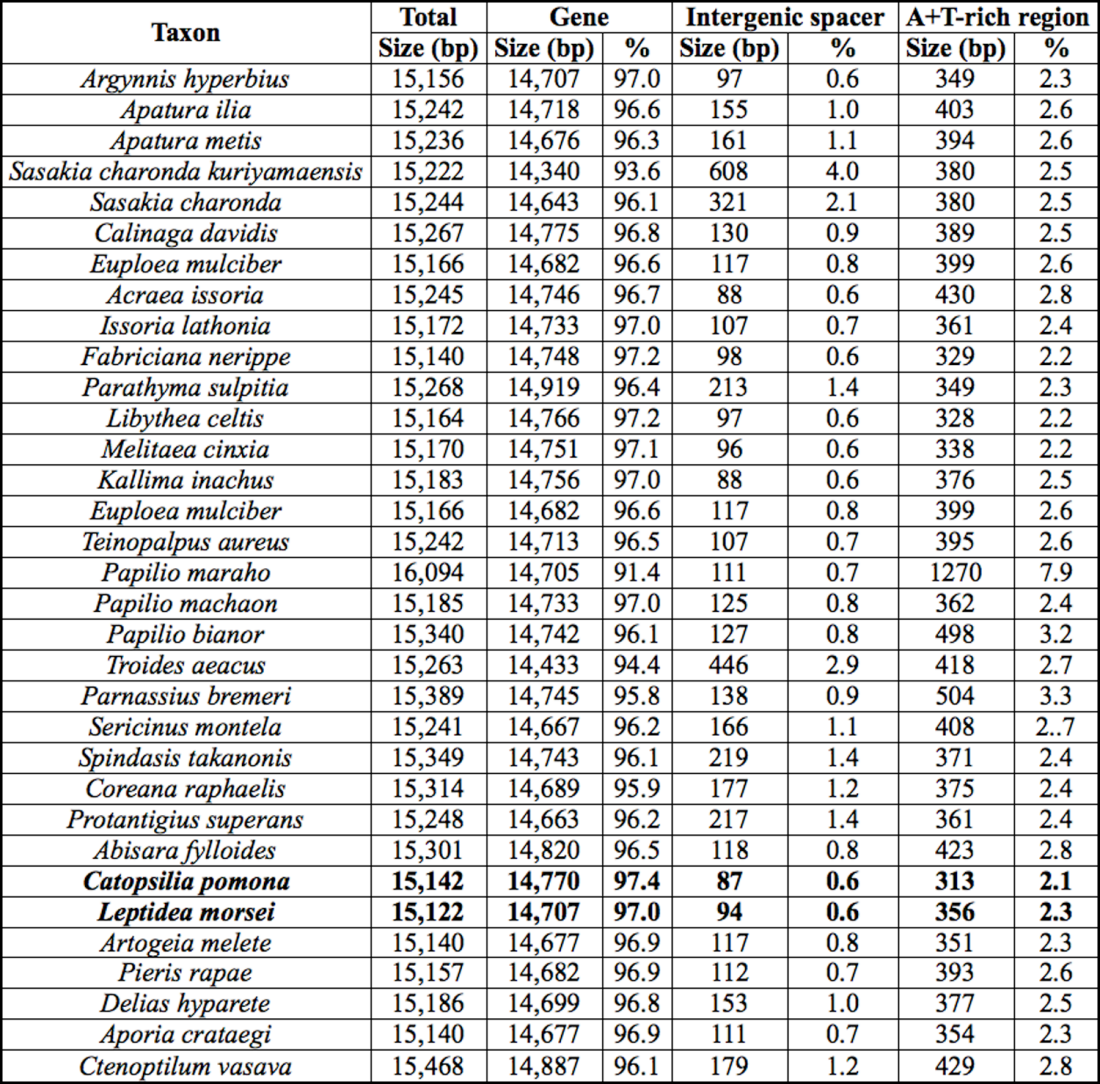
Size proportion of coding genes, intergenic spacers, and the A+T-rich region to the whole genome of the butterflies in this study.

The nucleotide compositions of the two entire mitogenome sequences were biased significantly toward A and T ( Table 5 ). These A+T contents were generally consistent with those of other butterfly mitogenomes, which ranged from 79.1% in *Eumenis autonoe* Esper (Lepidoptera: Nymphalidae) ( Kim, M. J. et al. 2010 ) to 82.7% in *Coreana raphaelis* Oberthür (Lepidoptera: Lycaenidae) ( Kim, I. et al. 2006 ). The base composition bias of an individual strand can be described by A+T skewness, caculated by (A%-T%)/(A%+T%), and G+C skewness, calculated by (G%—C%)/(G%+C%). The A+T and G+C skewness values in majority strands were calculated to be -0.122 and -0.121, respectively, for *L. morsei* , and -0.119 and -0.087, respectively, for *C. pomona.*

**Table 5.**
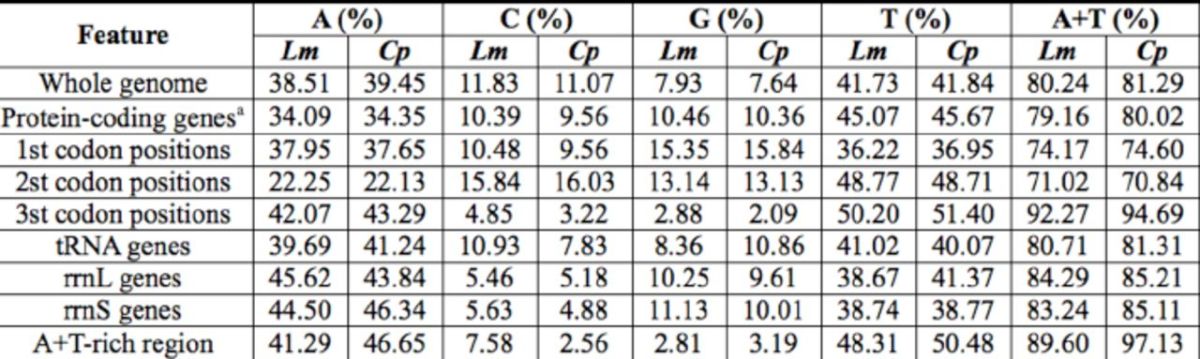
Nucleotide compositions in *L. morsei* ( *Lm* ) and *C. pomona* ( *Cp* ).

*a* Stop codons excluded.

### Protein-coding genes

All the protein-coding genes in *L. morsei* and *C. pomona* started with a typical ATN codon, with the only exception represented by the CGA start codon of the *COI* gene. For *L. morsei,* three genes *(ND2, ND5, ND6)* started with ATT, one *(ATP8)* with ATC, two *(ND3, ND4)* with ATA, and six *(ATP6, ND1, COII, COIII, ND4L, CytB)* with ATG. In comparison with *L. morsei, ATP8, ND3,* and *ND6* in *C. pomona* possessed different start codons, namely ATT, ATT, and ATC, respectively. These start codons were well-conserved in the sequenced butterfly mitogenomes. For instance, the *ND2* gene usually used ATT as the start codon, whereas the *COII* and *ATP6* genes frequently used ATG as the start codon ( Table 6 ).

**Table 6.**
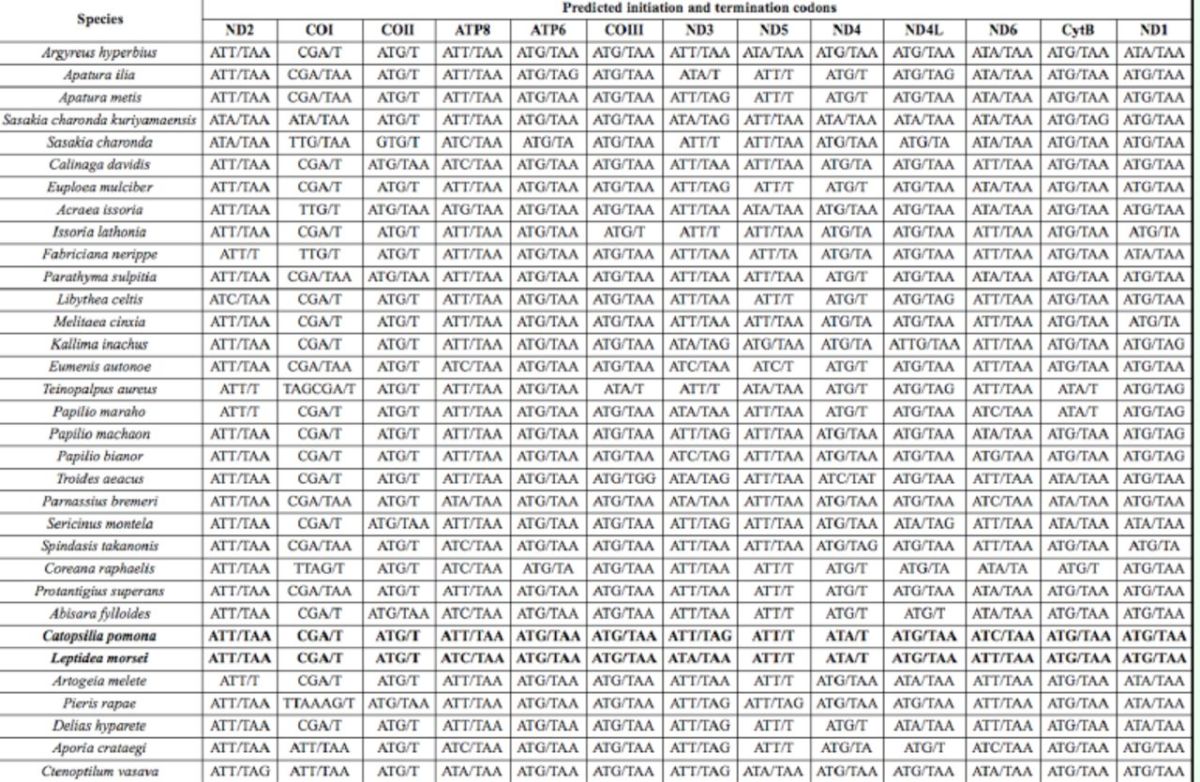
The 13 protein-coding gene initiation and termination codons in the mitogenomes of the 33 butterfly species in this study.

Nine protein-coding genes were terminated with the standard stop codon TAA, whereas the *COI, COII, ND4,* and *ND5* genes used T as a truncated stop codon. The only difference in stop codons between the two pierid species was found in the *ND3* gene, that is, *L. morsei* used TAA instead of TAG, which appeared in *C pomona.* Furthermore, incomplete stop codons were detected frequently in the *COI, COII,* and *ND5* genes in most insects, including all sequenced butterfly species ( Table 6 ). Incomplete stop codons would produce functional stop codons after polycistronic transcript cleavage and polyadenylation ( Ojala et al. 1981 ).

**Figure 2.**
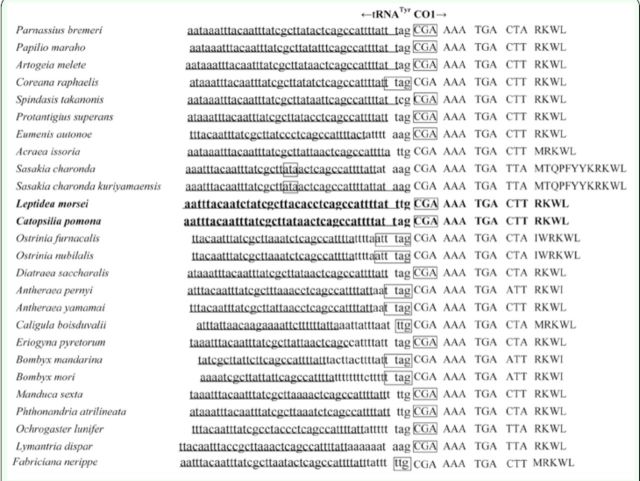
Alignment of the initiation codons of the *COI* genes of lepidopterans, including those of *L morsei* and *C. pomona.* The first four or five codons for *COI* and their amino acids are shown on the right-hand side of the figure. Underlined nucleotides indicate the adjacent partial sequence of *tRNA ^T^ y ^r^ .* Arrows indicate the transcriptional direction. Boxed nucleotides indicate the currently proposed translation initiators for the *COI* gene of lepidopteran insects. The start codon for *L morsei* and *C. pomona* is designated as CGA. High quality figures are available online.

Previous studies reported that most lepidopterans used the codon CGA as the start codon for *COI* ( Fig. 2 ). However, exceptions have been reported; for example, TTG was proposed as the start codon in *Acraea issoria* Hübner (Lepidoptera: Nymphalidae) ( Hu et al. 2010 ), *Caligula boisduvalii* Eversmann (Lepidoptera: Saturniidae) ( Kim, I. et al. 2006 ), and *Fabriciana nerippe* Felder (Lepidoptera: Nymphalidae) ( Kim, M. J. et al. 2011a ); ATT in *A. crataegi* ( Park et al. 2012 ) and *Ctenoptilum vasava* Moore (Lepidoptera: Hesperiidae) ( Hao et al. 2012 ); TTAG in *Bombyx mori* L. (Lepidoptera: Bombycidae) ( Yukuhiro et al. 2002 ) and *C raphaelis* ( Hong, G. Y. et al. 2009 ); and ATTTAG in *Ostrinia nubilalis* Hübner and *Ostrinia furnacalis* Guenée (Lepidoptera: Crambidae) ( Coates et al. 2005 ). In this study, a typical ATN initiator for *COI* in *L. morsei* and *C pomona* was not detected at their starting sites. The putative ATT start codon is commonly located upstream of the *COI* gene and frequently followed immediately by the TAG or TAA stop codon; thus, the CGA, not ATT, probably acted as the start codon here as in most butterflies.

Excluding stop codons, the A+T contents of protein-coding genes in *L. morsei* and *C pomona* were 79.16% and 80.02% ( Table 5 ), respectively, and these values were similar to those detected in other butterflies, which ranged from 76.8% in *E. autonoe* ( Kim, M. J. et al. 2010 ) to 81.5% in *C raphaelis* ( Kim, I. et al. 2006 ) ( Table 2 ). When the first, second, and third codon positions were considered separately, the highest A+T contents were in the third positions for *L. morsei* and *C pomona.* In addition, the highest T contents were detected in the second positions, and the lowest G contents in the third positions ( Table 5 ).

Exclusive of the stop codon, 3,713 and 3,724 amino acids were encoded by the mitogenomes of *L. morsei* and *C. pomona,* respectively ( Table 2 ). The amino acid numbers were well within the size range of 3,586 in *Sasakia charonda kuriyamaensis* Shirozu (Lepidoptera: Nymphalidae) (Hakozaki et al., unpublished, GenBank accession number NC_014223.1) to 3,740 in *Abisara fylloides* Moore (Lepidoptera: Riodinidae) (Shi et al. unpublished, College of Life Sciences, Anhui Normal University, China) detected in other butterflies. Among the amino acids, UUU (Phe), UUA (Leu), AUU (Ile), AUA (Met), and AAU (Asn) were the most frequently used codons in *L. morsei* and *C. pomona* ( Table 7 ), and similar codons were found in other butterfly species, such as *C. vasava* ( Hao et al. 2012 ) *, A. crataegi* ( Park et al. 2012 ), and *Sericinus montela* Gray (Lepidoptera: Papilionidae) ( Ji et al. 2012 ) *.*

**Table 7.**
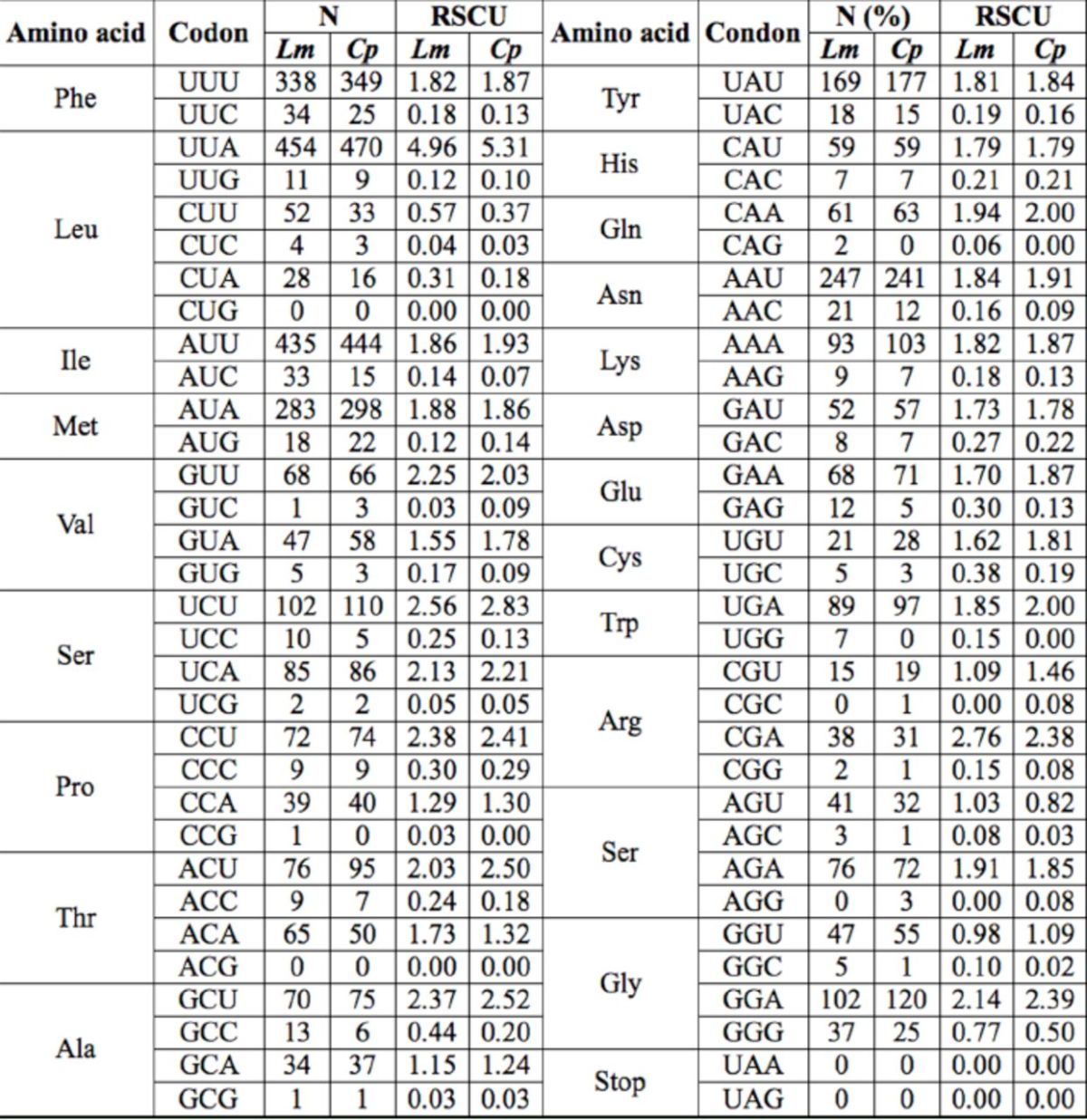
The codon usage in the mitogenomes of *L. morsei* ( *Lm* ) and *C. pomona* ( *Cp* ).

Start and stop codons excluded from total codon counts; N, frequency of codon use; RSCU, relative synonymous codon usage.

### Transfer RNAs and ribosomal RNAs

There were 22 tRNA genes (two each for serine and leucine, and one for each of the other amino acids) identified within the two pierid mitogenomes. An additional tRNA-like sequence ( *tRNA ^Leu^* [UUR]) located within the *16S rRNA* gene was detected in the mitogenome of *C. pomona* . The 22 tRNA genes ranging in size from 60 to 71 bp were interspersed throughout the two whole mitogenomes. The total sizes of the *L* . *morsei* and *C. pomona* tRNAs were 1,416 and 1,446 bp, respectively, with 80.68 and 81.05% A+T contents, respectively. All tRNAs could be folded into the typical clover leaf secondary structure, whereas *tRNA ^Ser^* (AGN) in both mitochondrial genomes lacked the dihydrouridine (DHU) loop. This feature has been shown in the majority of metazoan mitogenomes, including all those sequenced from butterflies ( Kim, I. et al. 2006 ; Hong, G. Y. et al. 2009 ; Hu et al. 2010;Kim, M. I. et al. 2009 ; Xia et al. 2011;Wang et al. 2011; Kim, M. J. et al. 2010, 2011a, 2011b; Chen et al. 2012; Shi et al. 2012; Tian et al. 2012 ). The anticodon for *tRNA ^Ser^* (AGN) in mitogenomes of butterfly species was either TCT, GCT, or ACT, whereas only GCT was detected in all other sequenced mitogenomes of pierid species ( *P. rapae, A. crataegi* , *D. hyparete* , and *A. melete* ) ( Hong, G. Y. et al. 2009 , Mao et al. 2010 , Park et al. 2012 , Shi et al. 2012 ). The tRNA-like structure ( *tRNA ^Leu^* [UUR]) was detected in the *16S rRNA* gene of *C. pomona* , and a similar observation had been made in *C. vasava* ( Hao et al. 2012 ). Interestingly, the 81 bp insertion of the tRNA-like sequence was made up completely of A and T, without G and C nucleotides. The *L. morsei* and *C. pomona* anticodon sequences of each tRNA isotype were identical to those of all other sequenced butterfly mitogenomes. As in other insects, unmatched base pairs were also detected in the stems of tRNAs. For *L. morsei* , there were 32 unmatched base pairs, consisting of 25 G-U, one A-A, and six U-U mismatches, whereas in *C. pomona* , 21 G-U, two A-A, and six U-U mismatches were identified.

Both of the two pierid mitogenomes harbored a large and a small ribosomal RNA subunit ( *16S rRNA* and *12S rRNA* ), located between *tRNA ^Leu^* (CUN) and *tRNA ^Val^* , and between *tRNA ^Val^* and the A+T-rich region, respectively. The length of the *16S rRNA* and *12S rRNA* genes in *L. morsei* were 1,337 and 764 bp, respectively, with A+T contents of 84.29 and 83.25%, respectively; the *16S rRNA* and *12S rRNA* in *C. pomona* were 1,332 and 779 bp in length, respectively, with A+T contents of 85.21 and 85.11%, respectively ( Table 2 ).

### Intergenic spacers and overlapping sequences

The mitogenomes of *L. morsei* and *C. pomona* harbored 11 and 15 intergenic spacers, ranging from 1 to 39 bp (94 bp in total) and 1 to 24 bp (87 bp in total), respectively ( Table 3 ). Among these, only three intergenic spacers were longer than 10 bp in both pierid species ( Table 3 ). The longest intergenic spacers, located between the *tRNA ^Gln^* and *ND2* genes in *L. morsei* and *C. pomona* , were 39 and 24 bp in length, respectively, with A+T contents of 90.35 and 91.67% respectively. This spacer is present in all of the butterfly mitogenomes sequenced to date, whereas absent in all non-lepidopteran insects. Another long spacer harboring the 7 bp ATACTAA motif, located between the *tRNA ^Ser^* (UCN) and *ND1* genes, has been observed commonly in most insect groups including all other butterflies.

In addition, there were 33 overlapping nucleotides scattered over 13 locations in *L. morsei* , and 26 nucleotide overlaps scattered over eight locations in *C. pomona* ( Table 2 ). Among these overlaps in the two pierid species, the longest one was 8 bp in length and located between *tRNA ^Trp^* and *tRNA ^Cys^* with the 7 bp motif AGCCTTA; the second longest one was 7 bp in length and located between *ATP8* and *ATP6* with the 7 bp motif ATGATAA. Both of these motifs have been observed in the mitogenomes of many butterfly species, including all the other pierids sequenced.

### The A+T-rich region

**Figure 3.**
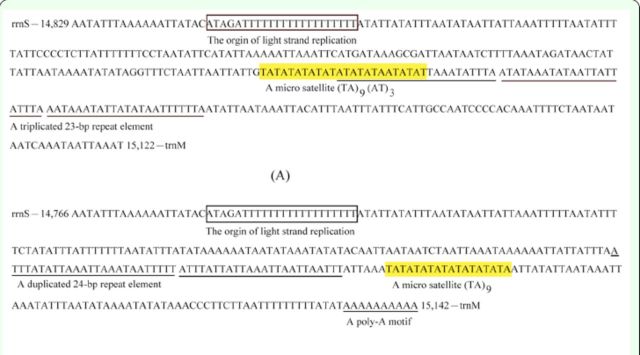
Structures of the A+T-rich regions in the mitogenomes of *L morsei* (A) and *C. pomona* (B). High quality figures are available online.

The A+T-rich regions of *L. morsei* and *C. pomona* were 356 and 313 bp in length, respectively, with A+T contents of 89.60 and 97.13%, respectively. Among the A+T-rich regions of all the butterfly mitogenomes sequenced, that of *C. pomona* was the shortest in length and the highest in A+T content ( Table 2 ). The A+T-rich regions of *L. morsei* and *C. pomona* contained the motif ATAGA, followed by a 19 and 18 bp poly-T stretch, respectively ( Fig. 3 ). Besides, the regions also included microsatellite-like elements, such as (TA) _9_ (AT) _3_ in *L. morsei* and (TA) _9_ in *C. pomona* , which were preceded by the ATTA motif characteristic of lepidopteran mitogenomes. Additionally, a triplicated 23 bp and a duplicated 24 bp repeat element of unknown function were found in *L. morsei* and *C. pomona* , respectively ( Fig. 3 ), and similar repeat elements were detected in other butterflies, such as *A. melete* ( Hong, G. Y. et al. 2009 ), *E. autonoe* ( Kim, M. J. et al. 2010 ), and *Agehana maraho* (Shiraki and Sonan) (Lepidoptera: Papilionidae) (syn. *Papilio maraho* ) ( Wu et al. 2010 ).

### Phylogenetic analysis

Several competing hypotheses exist on the phylogenetic family relationships in butterflies. Ehrlich (1967) , Ehrlich and Ehrlich (1967) , and Scott (1985) demonstrated the close relationship between the Nymphalidae and Lycaenidae and that between the Pieridae and Papilionidae via numerical taxonomic methods and morphological characters. Son and Kim (2011) and Wahlberg et al. (2005) obtained results consistent with the traditional view of the sister relationship between the Pieridae and the Nymphalidae + Lycaenidae group, with Papilionidae as the basal lineage, in agreement with the earlier hypothesis of Kristensen (1976) and supported by several recent studies ( Akaike 1974 , Kristensen 1976 , de Jong et al. 1996 , Ackery et al. 1999 , Wahlberg et al. 2005, Liao et al. 2010 , Kim, M. I. et al. 2010 , Kim, M. J. et al. 2011b, Son et al. 2011). However, Chai et al. (2012) and Zhang et al. (2012) , on the basis of mitochondrial genomic data, proposed a close relationship between the Pieridae and Lycaenidae, with the Nymphalidae being the sister group, in agreement with the hypothesis of Robbins (1988). Therefore, controversy exists regarding the relationships among the Nymphalidae, Pieridae, and Lycaenidae.

**Figure 4.**
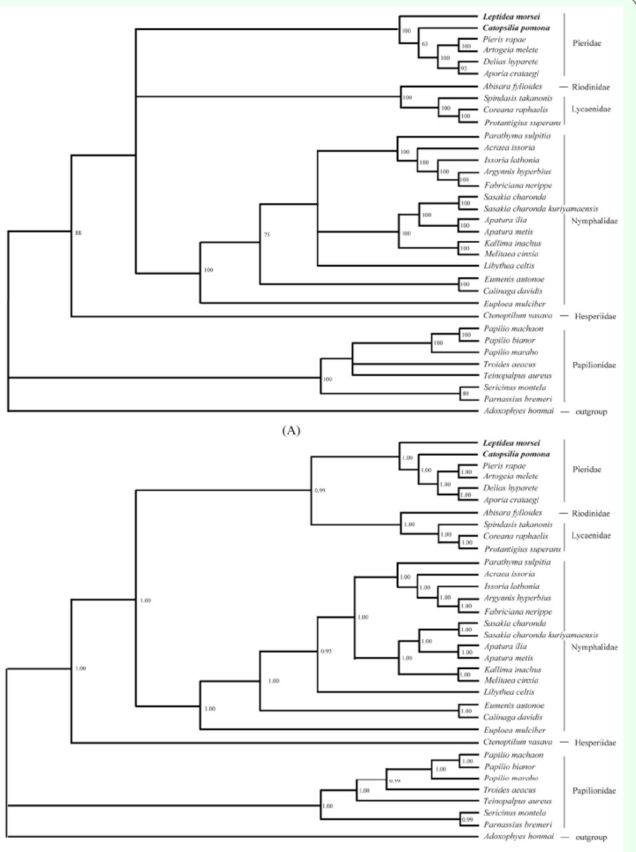
Phylogenetic trees of the butterflies in this study based on the nucleotide sequences of 13 protein-coding genes. (A) Maximum likelihood tree. (B) Bayesian inference tree. Numbers at each node indicate bootstrap percentage of maximum likelihood analysis and posterior probability of Bayesian inference analysis. High quality figures are available online.

In this study, we conducted phylogenetic analyses via Bayesian inference and maximum likelihood methods, using concatenated nucleotide datasets of 13 protein-coding genes (6,582 aligned sites, 910 gaps, and 3,746 excluded positions), resulting in similar tree topologies of the butterfly families Papilionidae, Pieridae, Lycaenidae, and Nymphalidae ( Fig. 4 A and B). The presented trees showed two major clusters ( Fig. 4 A and B). The first one had the Papilionidae as the basal lineage, and the other one included the rest of the butterfly families. Maximum likelihood and Bayesian inference trees suggest a close relationship between Pieridae and Lycaenidae ( Fig. 4 A and B), in agreement with the prevailing phylogeny of butterfly families (Hesperiidae (Papilionidae (Nymphalidae (Pieridae, Lycaenidae)))). Although the close relationship of the Pieridae and Lycaenidae proposed herein is contradictory to the traditional view (Pieridae (Nymphalidae, Lycaenidae)), the result is consistent with those of recent studies ( Kim, M. J. et al. 2010 , Chai et al. 2012 , Hao et al. 2012 , Zhang et al. 2012 ). However, uncertainty does exist regarding the sister relationship of the Pieridae and Lycaenidae as shown in the maximum likelihood tree ( Fig. 4A ). We also note that this relationship has been derived mainly from the protein-coding genes of the mitochondrial genome of butterflies.

In conclusion, although it may still be immature to suggest that the phylogeny of butterfly families is resolved, we do suggest that the sister relationship of the Pieridae and Lycaenidae is supported at the mitogenomic level in this study. We believe that the problem of butterfly phylogeny should be resolved step by step as gene sequence data and morphologic characters are being accumulated, and hopefully more sophisticated analytic tools will become available.
